# Virtual reality exposure training for music performance anxiety under pressure: a cross-cultural study of Chinese and Japanese music students

**DOI:** 10.3389/fpsyg.2026.1847383

**Published:** 2026-05-29

**Authors:** Mingyi Zhang, Xiao Chen, Yuanchun Jiang, Xue Wang, Xue Ma, Xiaoming Shan

**Affiliations:** 1 Qiqihar University, Qiqihar, China; 2 Jiangsu Second Normal University, Nanjing, China; 3 The First Hospital of Qiqihar, Qiqihar, China; 4 Beijing Huaxia Siyuan Technology Development Co., Beijing, China; 5 Graduate School of Music, Tokyo University of the Arts, Tokyo, Japan

**Keywords:** cross-cultural differences, music performance anxiety, performance evaluation contexts, response desynchrony, virtual reality training

## Abstract

Music performance anxiety (MPA) is prevalent among music students in high-pressure performance contexts. Virtual reality (VR)–based exposure training has been proposed as a potential intervention; however, its effectiveness across cultural contexts remains unclear. This study examined whether the impact of VR exposure training on MPA is moderated by cultural background. Thirty university-level clarinet majors were recruited from Tokyo University of the Arts (Japan; *n* = 16) and Qiqihar University (China; *n* = 14). Participants were stratified by baseline music performance anxiety (K-MPAI) and randomly assigned to a VR training group or a non-VR control group. Over a five-day training period, the VR group practiced in an immersive virtual performance-examination environment, while the control group followed conventional practice. Measures included pre-performance state anxiety (STAI-Y1), subjective distress (SUDS), heart rate, post-performance subjective evaluation, and adjudicated performance scores. Results revealed significant interaction effects between training condition and cultural background for both pre-performance state anxiety and post-performance subjective unease. Participants from the Chinese sample who received VR training reported lower levels of subjective anxiety relative to the non-VR condition, whereas comparable differences were not observed in the Japanese sample. No significant VR effects were observed for heart rate or performance scores. Correlation and regression analyses indicated relatively strong associations among subjective anxiety measures, whereas relationships between psychological, physiological, and performance indicators were generally weak. These findings provide preliminary evidence that responses to VR-based exposure training for MPA may vary across cultural and educational contexts and highlight the importance of context-sensitive applications of immersive technologies in music education.

## Introduction

In the context of globalization and the post-pandemic educational landscape, music education has increasingly been recognized as playing a role that extends beyond the acquisition of technical skills. A growing body of research suggests that systematic engagement in music learning may support cognitive development, emotional understanding, and social functioning among children and adolescents (Blasco-Magraner et al., 2021). Although much of this literature has focused on children and adolescents, the present study focuses specifically on university-level music majors engaged in professional training contexts. These potential benefits, however, may not manifest uniformly across educational and developmental contexts, as they depend on training demands, performance requirements, and evaluative environments. Sustained musical training has also been associated with the development of psychological resilience and a growth-oriented mindset, which may enhance students’ motivation and socio-emotional competencies (Holochwost et al., 2021). For students in professional music programs, supportive pedagogical relationships may further contribute to subjective well-being and mental health, particularly in learning environments characterized by high expectations and frequent evaluation (Bonneville-Roussy et al., 2020). However, the COVID-19 pandemic substantially disrupted conventional modes of music instruction, accelerating the adoption of digital and immersive technologies as institutions sought to maintain instructional quality and student engagement (Anderson et al., 2024; Ma and Wang, 2025). While music education has been widely associated with a range of cognitive, emotional, and social benefits, these advantages do not necessarily translate into positive experiences in all performance contexts. In particular, formal performance situations, such as examinations and recitals, often involve high evaluative pressure, may give rise to music performance anxiety (MPA) (Ilari and Cho, 2023).

Although music students have these benefits that are well documented, many continue to face the long-term psychological stress both in training and performance contexts with music performance anxiety (MPA) remaining a prominent concern. Other researchers have further established that MPA has been associated with a host of psychophysiological reactions such as increased heart rate and muscular tension and distraction and loss of confidence (Moreno-Gutiérrez et al., 2023; Irie et al., 2023; Twitchell et al., 2025). These reactions are likely to destabilize the performances and when they happen regularly, may undermine motivation to learn and the future career development. MPA is generally understood as an isolated cause, since it is typically attributed to the interaction of internal factors, such as fear of failure, perfectionism, self-criticism, and external factors that concern the performance environment, audience, teaching demands, and cultural expectations. Longitudinal research suggests that performance anxiety usually emerges at the start of musical education and tends to become more severe as musicians face greater technical requirements and choose their professional specialization. The findings show how this anxiety affects musicians’ mental health and their ability to sustain their professional careers (Casanova et al., 2025).

Performance under pressure is widely understood to involve a non-linear relationship between stress and task execution. The Yerkes–Dodson law describes this relationship as an inverted U-shaped function, in which moderate levels of arousal may support performance, whereas excessive stress compromises cognitive control and execution efficiency (Yerkes and Dodson, 1908). Across multiple research domains, empirical evidence has indicated that both acute and sustained stress can disrupt executive functioning, reduce working-memory efficiency, and alter autonomic nervous system activity, collectively undermining performance stability in complex tasks (Feng et al., 2021; Nuamah, 2024). Musical performance may be particularly vulnerable to such stress-related effects, given its reliance on the simultaneous coordination of fine motor control, emotional regulation, and sustained attentional focus. When stress levels exceed an optimal range, performers may experience rhythmic instability, intonation inaccuracies, or motor rigidity, processes that are often compounded by negative self-evaluation and anticipatory anxiety.

Different approaches have been recommended to deal with or treat music-based performance anxiety and they include cognitive behavioral therapy and mindfulness-based approaches as well as relaxation training which have shown varying degrees of success in research. The modified thinking patterns of performers who engage in these approaches may contribute to enhanced emotional stability yet these interventions are commonly administered under conditions of minimal performance pressure. These approaches may not achieve their full effectiveness since they may not fully address physiological arousal and anxiety responses which are crucial during actual performance-based evaluation situations.

In this regard, virtual reality (VR) has received increasing attention as a complementary intervention approach. Through immersive and controllable simulations of performance environments, VR allows for the possibility of expose performers repeatedly to stage-related stressors under adjustable conditions. Existing research suggests that VR-based stage simulations can increase familiarity with performance contexts and, in some cases, reduce subjective anxiety through graded exposure; however, reported effects remain preliminary and appear to depend on task characteristics and contextual factors (Bissonnette et al., 2016; Davis et al., 2020; Barrett et al., 2024; Bellinger et al., 2023). From a theoretical perspective, VR-based interventions can be understood as a form of controlled exposure, in which repeated interaction with performance-related stressors may facilitate habituation and reduce anxiety responses over time (Takac et al., 2019; Reeves et al., 2021). In addition, the sense of presence and ecological realism afforded by immersive environments may enhance the psychological relevance of the simulated context, thereby increasing the effectiveness of exposure-based processes (Shahid et al., 2024).

The effectiveness of VR-based interventions cannot be assumed to be uniform across cultural contexts. From a cross-cultural psychological perspective, such differences can be understood in relation to broader frameworks such as collectivism and culturally shaped achievement motivation, which influence how individuals interpret evaluative situations and regulate performance-related stress (Miksza et al., 2016; Bao and Yu, 2025). Within this context, music education systems and prevailing social expectations play an important role in shaping how performance pressure is experienced and internalized by students. Within the Chinese context, music training is closely tied to competitive examination systems and performance-based evaluation, circumstances under which external judgment often becomes a salient source of anxiety (Bao and Yu, 2025).

By contrast, music education in Japan is more commonly situated within long-term ensemble participation and collective practice. In these settings, performance-related anxiety tends to be more strongly associated with internalized self-demands, perfectionistic tendencies, and concerns about social responsibility and group harmony (Hebert, 2012; Irie et al., 2023). While students in both contexts deal with performance-related stress, the sources and psychological mechanisms driving this anxiety seem to vary in systematic ways. This aligns with the findings of Barros et al. (2024), who suggest that cultural and educational environments can moderate how individuals respond to performance-anxiety interventions. Such variations ultimately cast doubt on the cross-cultural generalizability of VR-based approaches.

Based on the theoretical considerations outlined above, including exposure-based mechanisms, culturally shaped responses to evaluative contexts, and the multidimensional nature of performance anxiety, the present study addresses the following research questions. The study investigates whether VR-based virtual stage training can reduce music performance anxiety among university-level music students in China and Japan. By examining both psychological and physiological responses across these two cultural contexts, the study addresses three related issues: first, the overall effectiveness of VR-based training in alleviating MPA; second, potential cross-cultural differences in anxiety responses within virtual stage environments; and third, whether culturally embedded sources of performance pressure moderate the effects of VR-based intervention. Through this cross-cultural approach, the study seeks to clarify the conditions under which VR may function as a viable tool for anxiety regulation in music education. It should be noted, however, that music performance anxiety is not a condition that can be entirely eliminated, but rather one that can be managed or alleviated to support more adaptive performance experiences.

## Method

### Participants

Participants were undergraduate students with a professional music background, all of whom were clarinet majors. Recruitment was conducted primarily at the Tokyo University of the Arts in Japan and Qiqihar University in China. All participants had received formal instrumental training, defined as structured instruction within a conservatory or university-level music program, and had prior experience performing in formal stage settings, such as recitals, juried examinations, or institutional concerts.

A total of 30 students participated in the study, including 16 from the Tokyo University of the Arts and 14 from Qiqihar University. Participants ranged in age from 18 to 29 years (M = 19.88, SD = 1.86), and no restrictions were imposed with respect to gender. Before the experiment began, all participants completed a demographic questionnaire and provided written informed consent in accordance with institutional ethical guidelines. Following enrollment, participants were assigned to either a virtual reality training group (VR) or a non–virtual reality control group (NVR), as described in the Research Procedure section. Gender was also considered during group assignment to avoid obvious imbalance across conditions. This study was performed in line with the principles of the Declaration of Helsinki. Approval was granted by the Ethics Committee of Tokyo University of the Arts (2023/12/07/No. 2023-20).

Both institutions are higher education settings specializing in professional music training, with structured curricula that include individual instruction, ensemble participation, and performance-based assessment. Admission to these programs is competitive and based on performance examinations, and students are regularly required to participate in recitals and evaluative performance contexts as part of their training. Participants were recruited through in-person announcements within the respective institutions, targeting students who were actively preparing for formal performance examinations at the time of the study.

### Instruments

The performance experience of musicians in high-pressure evaluative settings cannot be fully characterized by anxiety intensity alone. In addition to nervousness, aspects such as performance stability, execution fluency, and performers’ confidence in their own performance contribute to the overall performance state. For this reason, the present study employed multiple questionnaire-based instruments to capture participants’ subjective psychological responses at different stages of the performance examination.

Specifically, the Kenny Music Performance Anxiety Inventory (K-MPAI), the State–Trait Anxiety Inventory (STAI), the Subjective Units of Distress Scale (SUDS), and the Tsukamoto Performance Questionnaire were used to assess music performance–specific anxiety, general anxiety, immediate subjective distress, and post-performance psychological experience, respectively. In addition, participants in the virtual reality group completed an immersion questionnaire to evaluate their perceived experience during VR training. Together, these measures formed the basis for comparing anxiety-related responses and subjective performance experiences across experimental conditions.

#### Kenny music performance anxiety inventory

The Kenny Music Performance Anxiety Inventory (K-MPAI) was developed by Kenny (2006) and has undergone subsequent refinement and psychometric validation, including confirmation of its factor structure (Kenny, 2023). The instrument assesses music performance anxiety as experienced by musicians in performance-related contexts and has been widely applied across diverse populations and research settings (Chang-Arana et al., 2018; Dias et al., 2022; Niering et al., 2023).

The K-MPAI gives a complete picture of music performance anxiety. It includes fear of mistakes and judgement of people, bad thinking and feelings during performance, and body stress during performance, and self-perceptions of performance achievement and success. In addition, the scale includes items reflecting more stable psychological tendencies associated with performance anxiety, allowing it to encompass both situational and trait-like aspects of MPA.

In the present study, the K-MPAI was used to assess participants’ baseline levels of music performance anxiety prior to the intervention and served as a control variable for group assignment. The instrument was not intended to measure momentary state anxiety during the experimental procedure; rather, it was used to account for pre-existing differences in performance anxiety and to enhance comparability between experimental groups.

#### State–Trait Anxiety Inventory (STAI)

The State–Trait Anxiety Inventory (STAI; Spielberger et al., 1983) is a widely used instrument for assessing anxiety and distinguishes between transient, situation-specific anxiety (state anxiety) and relatively stable individual differences in anxiety proneness (trait anxiety). The scale comprises two subscales: STAI-Y1 for state anxiety and STAI-Y2 for trait anxiety. Owing to this distinction, the STAI has been extensively applied in clinical, educational, and performance-related research to examine anxiety responses in evaluative and high-pressure contexts (Gómez-Íñiguez et al., 2021), including studies of music performance anxiety (Kenny et al., 2004; Umuzdas et al., 2019; Guyon et al., 2022).

In the present study, the STAI was used to differentiate participants’ baseline anxiety tendencies from their immediate state anxiety in the performance examination context. Participants completed the STAI-Y2 during the preparatory phase prior to the VR training intervention to index trait anxiety, whereas the STAI-Y1 was administered approximately 10 min before the performance examination to assess anticipatory state anxiety. This design enabled the concurrent consideration of stable anxiety characteristics and situationally induced anxiety responses when evaluating music performance anxiety and the effects of the VR-based intervention.

#### Subjective units of distress scale (SUDS)

The Subjective Units of Distress Scale (SUDS), originally introduced by Wolpe (1990), is a self-report measure designed to capture individuals’ immediate subjective experience of anxiety or distress. The SUDS is a numerical rating scale that allows the subjects to express their level of tension or discomfort at a certain moment, focusing on the present and the personal impression rather than the clinical evaluation (Kaplan and Smith, 1995). For this reason, the SUDS has been widely used in both clinical and experimental research to assess momentary emotional responses across diverse stress-inducing contexts.

Owing to its simplicity and sensitivity to short-term fluctuations, the SUDS is particularly well suited for tracking dynamic changes in subjective emotional states over time. Previous studies shows that periodic SUDS assessments may accurately record the instantaneous fluctuations of perceived strain in the course of challenging tasks and also when performing tasks (Dozier et al., 2020). In the context of music performance, SUDS ratings have been shown to increase under high-pressure conditions and to decrease following repeated exposure or training interventions, reflecting changes in subjective anxiety regulation (Bissonnette et al., 2016).

In the present study, the SUDS was employed as an index of immediate subjective tension before and around the performance examination. Its inclusion was intended to complement the K-MPAI and STAI, which primarily assess baseline or situational anxiety at broader temporal scales and are less sensitive to rapid emotional fluctuations during experimental procedures. The inclusion of SUDS helped the study track the momentary feelings of stress in the participants during the different stages of the experiment, and it also allowed the evaluation of instantaneous fear responses in more detail between the virtual reality training and the usual training condition.

#### Tsukamoto performance questionnaire

The Tsukamoto Performance Questionnaire was developed to assess musicians’ retrospective evaluations of their own performance and performance experience following a musical task. Unlike anxiety-focused instruments, the questionnaire is not intended to measure anxiety states per se; rather, it emphasizes performers’ subjective appraisal of their performance process and outcomes relative to their personal expectations.

The questionnaire has been used in prior research examining psychological processes associated with music performance, particularly in post-performance contexts, where it has served as an indicator of perceived performance quality, stability, and overall performance experience (Yoshie and Shigemasu, 2007). Its items address multiple aspects of post-performance experience, including emotional reactions and overall subjective evaluations of performance.

In the present study, the Tsukamoto Performance Questionnaire was used specifically to assess participants’ post-performance subjective evaluations following the examination. Only items directly related to post-performance evaluation were included, whereas items concerning bodily symptoms or immediate uneasiness during performance were excluded. This selective use allowed the questionnaire to function as a focused measure of post-performance experience while minimizing conceptual overlap with instruments assessing anxiety and tension. Accordingly, the questionnaire served as a complementary indicator for examining whether VR-based training influenced participants’ perceived performance quality and overall performance experience.

### Heart rate monitoring

In addition to self-report measures, heart rate was recorded as a physiological indicator of arousal during the performance examination. Heart rate data were collected using the pulse monitoring function of an automatic blood pressure monitor (A&D Medical, UA-621), enabling assessment of heart rate changes across performance-related phases.

Heart rate is commonly used as an index of autonomic nervous system activity and has been shown to increase under conditions of heightened stress or evaluative pressure, reflecting elevated sympathetic activation (Soliński et al., 2024). Accordingly, heart rate has been widely employed in performance-related research as an objective indicator of physiological arousal and autonomic activation (Gallego et al., 2022; Candia et al., 2023; Moreno-Gutiérrez et al., 2023).

In the present study, heart rate was measured before the performance examination and again after its completion. These data were examined alongside subjective measures, including the SUDS and the STAI-Y1/Y2, to allow comparison between physiological responses and self-reported psychological states. The aim of this multimodal approach was to characterize participants’ responses to the performance examination more thoroughly.

Previous research has further suggested that physiological arousal may be related to performance quality, with excessively elevated heart rate potentially interfering with motor control and attentional allocation, whereas moderate arousal may support sustained focus (Nakahara et al., 2010; Watanabe et al., 2025). Heart rate data were analyzed alongside post-performance subjective assessments, including the Tsukamoto Performance Questionnaire, to investigate the impact of VR-based training on tension responses at both psychological and physiological levels.

### Experimental equipment and environment

To create a performance examination setting with a high degree of ecological realism, the present study employed a combination of conventional musical instruments and virtual reality–based equipment. Conventional instruments were used for actual performance, whereas VR-related apparatuses supported the simulation of performance examination contexts.

The selection and configuration of the equipment reflected both the professional requirements of musical performance and the need for experimental control. This design allowed participants to perform in conditions closely resembling a real examination setting while ensuring that the data collected were suitable for systematic analysis.

#### Clarinet

**Figure 1:**
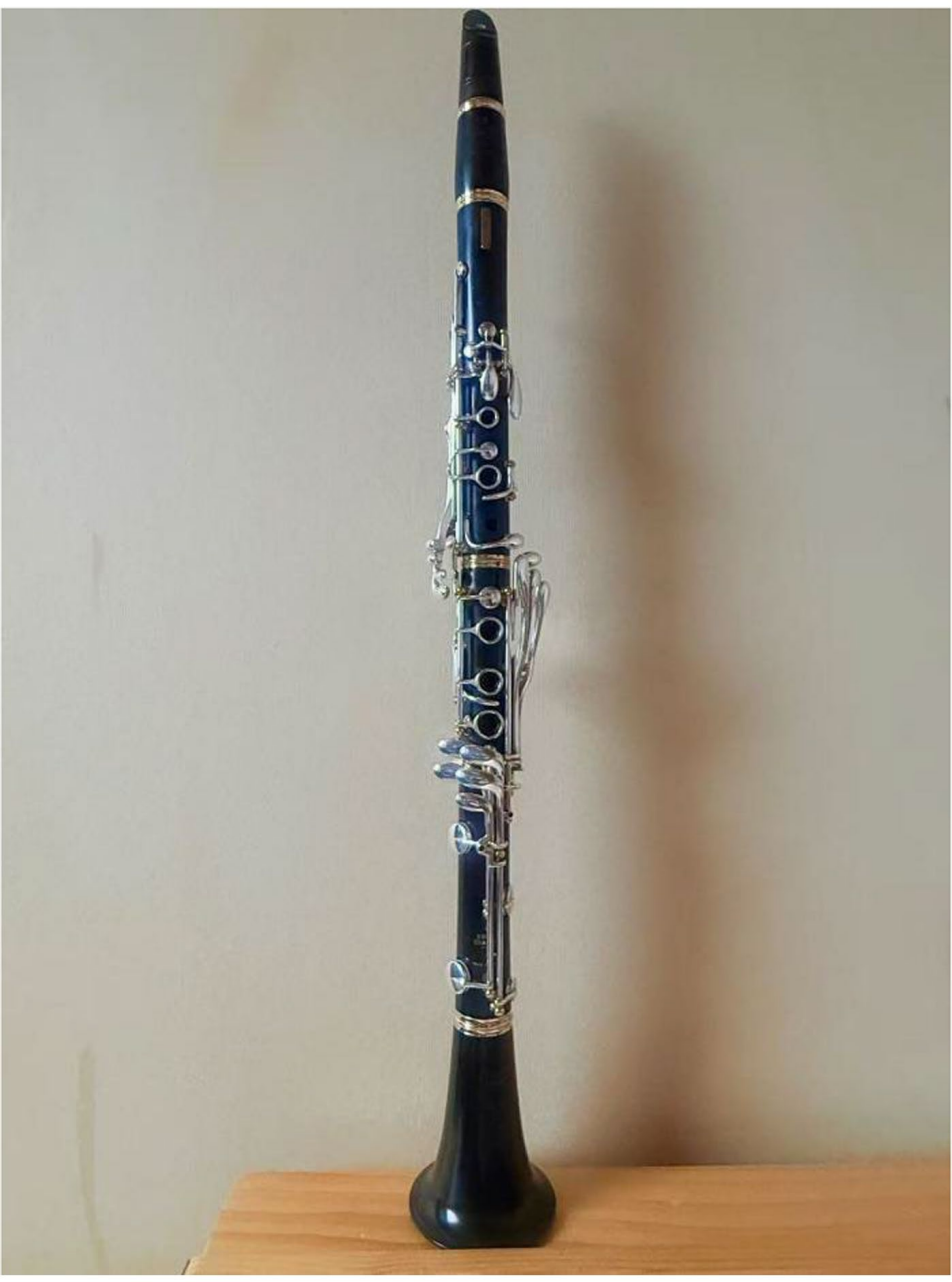
Clarinet used in the experimental performance task.

The clarinet (Figure 1) served as the primary performance instrument in the present study. Clarinet performance places high demands on breath control, embouchure stability, and fine finger coordination, rendering performance outcomes particularly sensitive to performers’ psychological states. Under high-pressure examination or stage conditions, anxiety-related responses may disrupt breathing patterns, embouchure control, and movement fluency, thereby affecting performance stability and intonation accuracy. For this reason, the clarinet provided an appropriate medium for examining the relationship between music performance anxiety and performance outcomes. The focus on clarinet players was also influenced by practical recruitment considerations and the need to maintain a relatively homogeneous instrumental context. Rather than suggesting that the clarinet represents a uniquely suitable case, it is treated here as one example within a controlled performance setting.

Because data collection was conducted in both Japan and China, consistent principles were applied to equipment configuration and experimental conditions across sites. Virtual reality–related equipment, performance task formats, and data collection procedures were kept identical or functionally equivalent to minimize potential confounding effects and to ensure the comparability of data obtained from the two locations.

All participants were clarinet majors, ensuring familiarity with the instrument and its performance demands. The instruments used in the experiment were provided by the researchers. Participants used their own reeds during the performance, reflecting their typical playing conditions.

#### Virtual reality equipment and environment

The virtual reality (VR) environment was shown using the Apple Vision Pro. This is a head mountable display that combines a high resolution visual output with spatial audio and simple interaction features to be an immersive performer of performance test scene presentation system. None of the participants had prior experience using VR equipment. A familiarization period with no time limit was provided prior to the training sessions, allowing participants to practice until they felt confident in operating the system.

The virtual performance examination environment was constructed using three-dimensional video footage recorded in actual performance venues and subsequently presented through the Apple Vision Pro. An Insta360 panoramic camera was employed to record 360-degree video footage of the examination settings, including the performer’s viewpoint, the evaluators’ locations, the audience arrangement, and the overall spatial atmosphere, in order to capture the performance context. The recorded footage was processed using Adobe Premiere Pro for basic editing and formatting, then exported in a three-dimensional format compatible with VR playback. Finalized video files were played on the Apple Vision Pro using Moon Player during the training phase.

**Figure 2:**
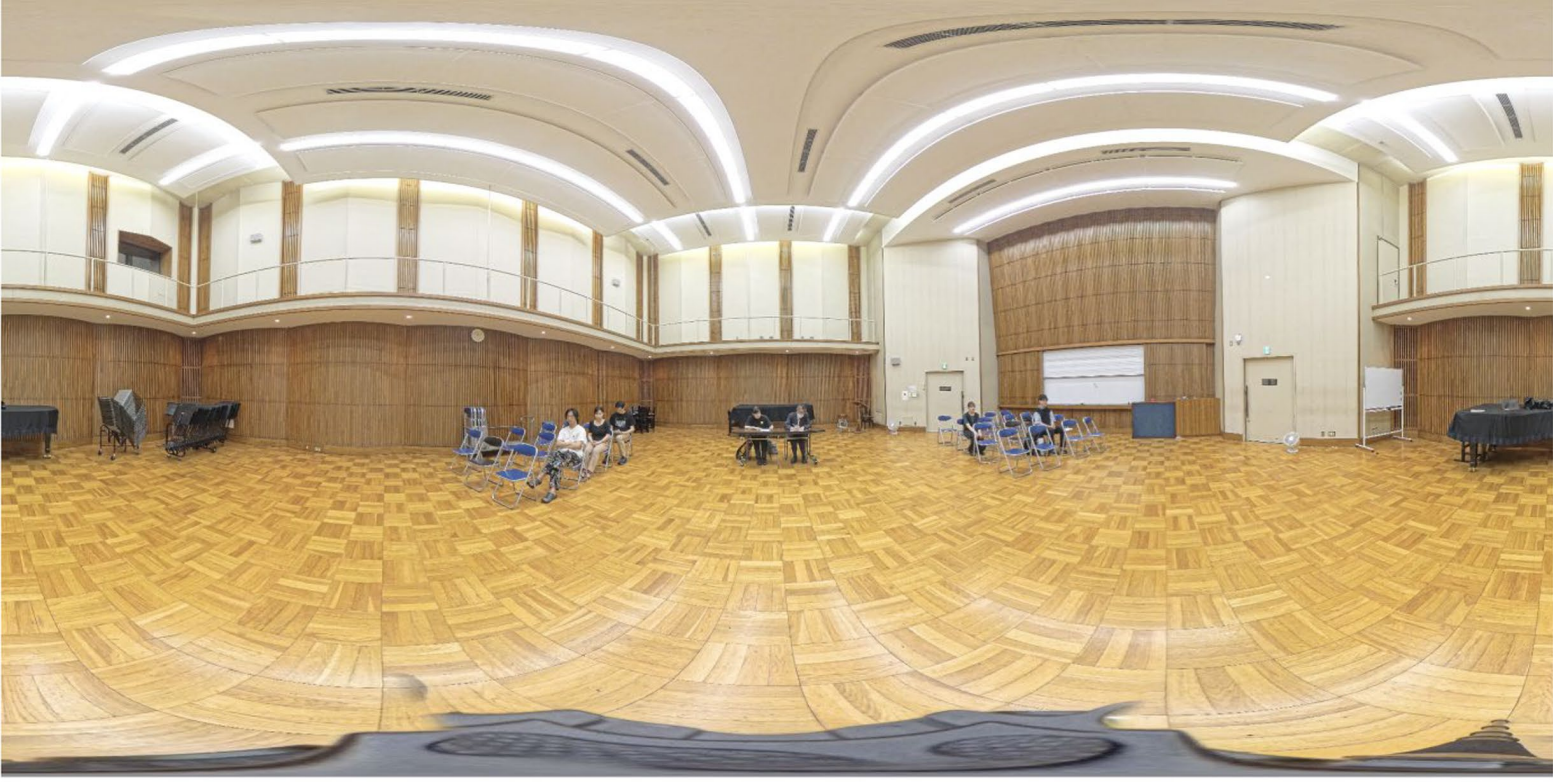
Example of the virtual performance examination scene viewed by participants through the Apple Vision Pro (Tokyo University of the Arts).

Because the VR environment was based on recordings from real performance venues, key spatial and visual characteristics—such as room scale, audience placement, and evaluator presence—closely approximated those of actual performance examinations. This design provided ecological realism, and participants were able to practically practice performance under the simulated experience of examination conditions (Gómez-Sirvent et al., 2024). An example of the virtual performance examination scene which the participants in the Tokyo University of the Arts watched is shown in Figure 2.

**Figure 3:**
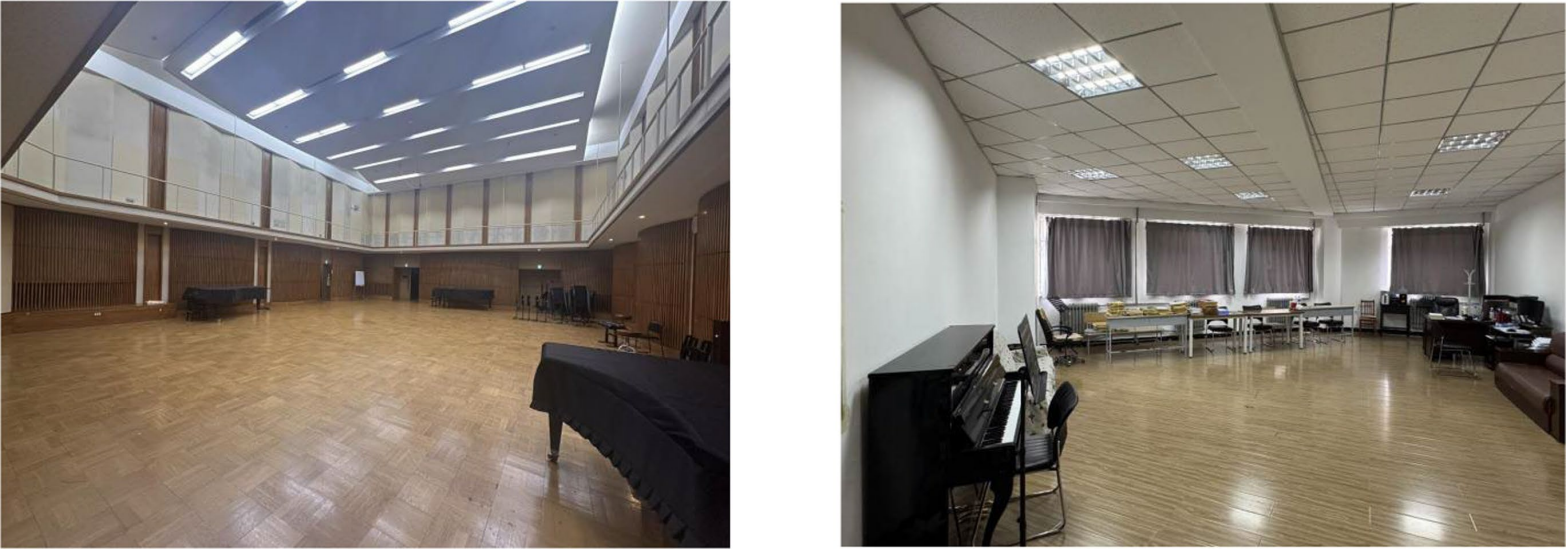
Experimental environments for the non-virtual reality (NVR) condition (left: Tokyo University of the Arts; right: Qiqihar University).

In the non–virtual reality (NVR) condition, performance examinations and data collection were conducted in physical venues at the Tokyo University of the Arts in Japan (Figure 3, left) and Qiqihar University in China (Figure 3, right). At both sites, participants completed the performance task in real examination settings without VR intervention.

At the Tokyo University of the Arts, the examination took place in a small concert hall with a stage configuration, measuring approximately 16.8 m × 13.98 m × 6.9 m and including fixed audience seating. At Qiqihar University, a multifunctional performance classroom was used, measuring approximately 9.5 m × 8.8 m × 3.0 m. Although smaller in scale, this space provided the essential facilities required for performance examinations (Figure 3).

To reduce potential environmental confounds, temperature, lighting, and external noise were kept as stable as possible at both venues during data collection. Performance tasks and data collection procedures followed the same experimental protocol across sites. Conducting the NVR condition in these physical performance spaces allowed the study to capture participants’ psychological and physiological responses in real examination environments, providing a reference for comparison with the virtual reality condition. To enhance comparability across sites, key environmental and procedural factors-including performance tasks, evaluation procedures, and data collection manner-were kept as consistent as possible, and environmental conditions such as lighting, temperature, and noise were controlled within practical constraints.

### Research procedure and experimental design

The research procedure comprised four stages: participant screening and group assignment, the training or practice phase, data collection prior to the performance examination, and data collection following the examination.

After participant screening was completed (see Section 2.1), group assignment was conducted. Because pre-existing anxiety levels may influence psychological and physiological responses in performance examination contexts, all participants first completed the Kenny Music Performance Anxiety Inventory (K-MPAI) to assess baseline music performance anxiety. Participants were then assigned to either the virtual reality training group (VR) or the non–virtual reality control group (NVR) using a stratified randomization procedure based on K-MPAI scores, ensuring comparable baseline anxiety distributions across groups. Gender was also taken into account to maintain a relatively balanced gender distribution and to reduce potential gender-related confounding effects. Following group assignment, participants completed the trait anxiety subscale of the State–Trait Anxiety Inventory (STAI-Y2) as an index of trait anxiety.

The study then entered the training phase, which followed a five-day schedule. Participants in the VR group engaged in approximately 20 to 25 min of VR-based performance training per day, practicing within a virtual performance examination environment. Participants in the NVR group completed conventional performance practice over the same period without VR equipment. The duration and daily training time were determined by practical constraints related to the course schedule in which the study was conducted. During this phase, participants completed only their assigned training or practice activities; no psychological questionnaires or formal physiological measurements were administered in order to avoid potential interference with the training process.

Upon completion of the training period, all participants proceeded to the formal performance examination. Approximately 10 min prior to the examination, participants completed the STAI-Y1 and the Subjective Units of Distress Scale (SUDS) to assess anticipatory state anxiety and immediate subjective tension. Heart rate (pulse rate, PR) was recorded concurrently as a physiological indicator. Performance materials were standardized across participants, and task difficulty was kept approximately equivalent to minimize potential confounding effects related to musical content.

Following the performance examination, participants completed the Tsukamoto Performance Questionnaire to provide a subjective evaluation of performance quality and overall performance experience. Heart rate monitoring continued after the examination to capture post-performance physiological recovery and to allow comparison with pre-performance measures. Performance scores awarded during the examination were also included as objective indicators in analyses examining the relationship between anxiety and performance outcomes. In addition, participants in the VR group completed a presence questionnaire assessing perceived immersion, realism, and attentional engagement during VR training.

The study employed a 2 × 2 between-subjects experimental design. Training condition (VR vs. NVR) and cultural background (Japan vs. China) served as independent variables. Dependent variables included subjective anxiety measures, physiological responses, performance evaluations, and presence-related measures for the VR group. This design allowed examination of the main effects of training condition and cultural background, as well as their interaction. Assumptions of normality and homogeneity of variance were assessed prior to conducting the ANOVA and were found to be adequately met. Moreover, effect sizes (partial η^2^) were interpreted following commonly used benchmarks (e.g., small = 0.01, medium = 0.06, large = 0.14).

## Results

### Participant characteristics

A total of 30 valid participants were included in the analysis, with 16 students recruited from Tokyo University of the Arts and 14 from Qiqihar University.

Participants from Tokyo University of the Arts had a mean age of 20.60 years (*SD* = 2.69, median = 20). On average, they had studied their instrument for 10.27 years (*SD* = 2.52, median = 10), practiced 3.87 days per week (*SD* = 0.35, median = 4), and practiced 3.20 h per day (*SD* = 0.77, median = 3). During the previous year, they reported a mean of 2.93 solo performances (*SD* = 1.03, median = 3) and participated in an average of 2.20 ensemble or chamber music performances (*SD* = 1.21, median = 2).

Participants from Qiqihar University had a mean age of 19.87 years (*SD* = 1.51, median = 19). Their average years of instrumental training were 4.40 years (*SD* = 2.13, median = 4). They reported practicing an average of 3.00 days per week (*SD* = 1.13, median = 4) and 1.60 h per day (*SD* = 0.51, median = 2). In the previous year, they reported an average of 1.80 solo performances (*SD* = 0.77, median = 2) and participated in 1.73 ensemble or chamber music performances on average (*SD* = 0.70, median = 2).

### Baseline music performance anxiety (K-MPAI)

To examine the comparability of baseline music performance anxiety between the virtual reality training group (VR) and the non–virtual reality control group (NVR), total scores on the Kenny Music Performance Anxiety Inventory (K-MPAI) were analyzed prior to the intervention.

The results indicated that the VR group (*M* = 52.60, *SD* = 10.41) and the NVR group (*M* = 51.87, *SD* = 7.78) exhibited similar levels of baseline music performance anxiety. An independent-samples t test revealed no significant difference between the two groups, *t* = 0.22, *p* = 0.829.

These findings suggest that the two groups were comparable in terms of initial music performance anxiety prior to the intervention, allowing subsequent group differences to be reasonably attributed to the training condition.

### State anxiety prior to the performance

Regarding state anxiety, the State–Trait Anxiety Inventory Form Y-1 (STAI-Y1), administered prior to the formal performance examination, was used as an index of participants’ immediate state anxiety in the performance examination context. A two-way analysis of variance (ANOVA) was conducted with training condition (VR vs. NVR) and school (Tokyo University of the Arts vs. Qiqihar University) as between-subjects fixed factors.

The results revealed a significant main effect of training condition (*F* = 4.97, *p* = 0.035), indicating that, overall, participants who received virtual reality training reported lower levels of pre-performance state anxiety than those in the non-virtual reality control group. In addition, a significant interaction effect between training condition and school was observed (*F* = 6.71, *p* = 0.016), suggesting that the effect of virtual reality training differed across the two institutional contexts.

To provide a clearer sense of the precision of the estimates, 95% confidence intervals (CIs) were also examined. For the sample of Qiqihar University, participants in the virtual reality training group showed lower STAI-Y1 scores (*M* = 36.52, 95% *CI* [32.20, 40.85]) than those in the non-virtual reality control group (*M* = 49.81, 95% *CI* [43.60, 56.02]). In contrast, for the sample of Tokyo University of the Arts, STAI-Y1 scores were largely similar across conditions, with the virtual reality group (*M* = 49.75, 95% *CI* [43.53, 55.97]) and the non-virtual reality group (*M* = 49.00, 95% *CI* [37.91, 60.09]) showing overlapping intervals.

*Post-hoc* simple effects analyses were conducted to examine the effect of training condition within each school. The results indicated that, among participants from Qiqihar University, STAI-Y1 scores were significantly lower in the virtual reality training group than in the non-virtual reality control group (*p* = 0.006). In contrast, no significant difference in STAI-Y1 scores between training conditions was observed among participants from Tokyo University of the Arts (*p* = 0.892) (see Table 1).

**Table 1:** Two-way ANOVA results for the effects of training condition and school on pre-performance state anxiety (STAI-Y1).

Source	df	SS	*F*	*p*	Partial *η^2^*
Training condition (VR vs. NVR)	1	530.52	4.97	0.035*	0.161
School (Tokyo vs. Qiqihar)	1	286.27	2.68	0.114	0.093
Training condition × School	1	717.01	6.71	0.016*	0.205
Residual	26	2775.78			

VR, virtual reality training; NVR, non-virtual reality control group. *p < 0.05.

### Subjective units of distress scale (SUDS)

For subjective distress, the virtual reality (VR) training group reported a lower mean SUDS score (*M* = 5.33, *SD* = 1.72) than the non-virtual reality (NVR) control group (*M* = 6.07, *SD* = 2.37). Mean SUDS scores were comparable across schools (Tokyo University of the Arts: *M* = 5.63, *SD* = 2.31; Qiqihar University: *M* = 5.79, *SD* = 1.85).

A two-way analysis of variance (ANOVA) revealed that the main effect of training condition did not reach statistical significance (*p* > 0.05), and the main effect of school was also non-significant (*p* > 0.05). However, the interaction between training condition and school showed a marginal trend toward significance (*p* = 0.087).

To give a clearer sense of the variability in these estimates, 95% confidence intervals (CIs) were also considered. For the sample of Qiqihar University, the virtual reality group showed slightly lower SUDS scores (*M* = 5.14, 95% *CI* [4.35, 5.94]) compared to the non-virtual reality group (*M* = 6.57, 95% *CI* [5.71, 7.43]). In contrast, within the sample of Tokyo University of the Arts, SUDS scores were quite similar across conditions, with the virtual reality group (*M* = 5.88, 95% *CI* [4.66, 7.09]) and the non-virtual reality group (*M* = 5.38, 95% *CI* [2.85, 7.90]) showing broadly overlapping intervals.

### Heart rate (HR)

With respect to physiological responses, heart rate (HR) measured before and after the performance examination was used as an objective physiological indicator to examine autonomic arousal during the performance examination context and to compare these responses with subjective anxiety measures. A two-way analysis of variance (ANOVA) was conducted on HR data with training condition (VR vs. NVR) and school (Tokyo University of the Arts vs. Qiqihar University) as between-subjects fixed factors.

For pre-performance HR, a significant main effect of school was observed (*p* = 0.011), indicating that, overall, participants from Qiqihar University exhibited higher pre-performance HR than those from Tokyo University of the Arts. The main effect of training condition and the interaction between training condition and school were not significant (*p* > 0.05). For post-performance HR, a significant interaction effect between training condition and school was found (*p* = 0.048), whereas the main effects of training condition and school did not reach statistical significance (*p* > 0.05). Post-hoc comparisons indicated that, within each school, no significant differences in post-performance HR were observed between the VR and NVR groups (*p* > 0.05). In addition, analyses using HR change scores (post-performance minus pre-performance) as the dependent variable revealed no significant main effects or interaction effects (*p* > 0.05).

To give a clearer sense of the variability in these estimates, 95% confidence intervals (CIs) were also examined. For pre-performance HR, the sample of Tokyo University of the Arts showed very similar values across conditions, with the virtual reality group (*M* = 85.13, 95% *CI* [80.26, 89.99]) and the non-virtual reality group (*M* = 84.88, 95% *CI* [76.07, 93.68]) showing substantial overlap. In the sample of Qiqihar University, the non-virtual reality group showed somewhat higher pre-performance HR (*M* = 105.57, 95% *CI* [97.60, 113.55]) than the virtual reality group (*M* = 92.62, 95% *CI* [85.05, 100.19]), although the intervals still overlapped. For post-performance HR, a broadly similar pattern was observed. In the sample of Tokyo University of the Arts, the virtual reality group had a mean of 99.00 (95% *CI* [89.79, 108.21]) and the non-virtual reality group had a mean of 104.50 (95% *CI* [99.88, 109.12]). In the Qiqihar University sample, the virtual reality group showed a mean of 95.81 (95% *CI* [87.22, 104.40]) and the non-virtual reality group a mean of 97.81 (95% *CI* [91.74, 103.88]). Overall, the confidence intervals suggest considerable variability and no stable separation between conditions within schools.

### Tsukamoto performance questionnaire

The results of the two-way analysis of variance (ANOVA) revealed a significant main effect of training condition (*F* = 4.28, *p* = 0.049), indicating that participants in the virtual reality (VR) training group reported significantly lower Tsukamoto performance anxiety scores than those in the non-virtual reality (NVR) control group. A significant main effect of school was also observed (*F* = 4.24, *p* = 0.05), showing that participants from Qiqihar University reported significantly lower Tsukamoto scores than those from Tokyo University of the Arts.

In addition, a significant interaction effect between training condition and school was found (*F* = 9.34, *p* = 0.005). Post-hoc simple effects analyses indicated that, among participants from Qiqihar University, the VR training group reported significantly lower Tsukamoto scores than the NVR control group (*p* = 0.002). In contrast, no significant difference between training conditions was observed among participants from Tokyo University of the Arts (*p* > 0.05) (Table 2).

**Table 2:** Two-way ANOVA results for the effects of training condition and school on Tsukamoto performance questionnaire scores.

Source	df	SS	*F*	*p*	Partial *η^2^*
Training condition (VR vs. NVR)	1	353.63	4.28	0.049*	0.141
School (Tokyo vs. Qiqihar)	1	350.17	4.24	0.050*	0.140
Training condition × School	1	772.21	9.34	0.005**	0.264
Residual	26	2148.95			

VR, virtual reality training; NVR, non-virtual reality control group.

*p < 0.05, **p < 0.01.

To give a clearer sense of the variability in these estimates, 95% confidence intervals (CIs) were also examined. For the Qiqihar University sample, the virtual reality group showed lower Tsukamoto scores (*M* = 63.33, 95% *CI* [59.88, 66.78]) compared to the non-virtual reality group (*M* = 74.14, 95% *CI* [69.62, 78.67]). In contrast, within the Tokyo University of the Arts sample, Tsukamoto scores were relatively similar across conditions, with the virtual reality group (*M* = 75.25, 95% *CI* [71.02, 79.48]) and the non-virtual reality group (*M* = 76.63, 95% *CI* [71.45, 81.80]) showing overlapping intervals.

### Performance examination scores

With respect to performance outcomes, adjudicators’ scores assigned after the formal performance examination were used as an objective indicator of performance quality. The results showed that mean performance scores were comparable between the virtual reality (VR) training group and the non-virtual reality (NVR) control group. In contrast, differences were observed between schools, with participants from Qiqihar University obtaining higher average scores than those from Tokyo University of the Arts.

A two-way analysis of variance (ANOVA) revealed a significant main effect of school (*F* = 11.37, *p* = 0.002), indicating that adjudicated performance scores were significantly higher for participants from Qiqihar University than for those from Tokyo University of the Arts. The main effect of training condition and the interaction between training condition and school were not statistically significant (*p* > 0.05).

### Correlation analysis

To examine the overall associations among virtual reality training, cultural background, and anxiety-related measures, Pearson correlation analyses were conducted to assess the relationships among pre-performance state anxiety (STAI-Y1), subjective distress (SUDS), heart rate change (HR delta), post-performance subjective anxiety (Tsukamoto Performance Questionnaire), adjudicated performance scores, training condition (VR vs. NVR), and cultural background (Qiqihar University vs. Tokyo University of the Arts).

The results indicated a significant positive correlation between STAI-Y1 and SUDS (*r* = 0.58, *p* < 0.001), as well as a strong positive correlation between STAI-Y1 and Tsukamoto scores (*r* = 0.82, *p* < 0.001), suggesting a consistent association between pre-performance state anxiety and post-performance subjective anxiety. In addition, SUDS was moderately positively correlated with Tsukamoto scores (*r* = 0.48, *p* = 0.007).

In contrast, heart rate change was not significantly correlated with STAI-Y1 (*r* = −0.02, *p* = 0.94), SUDS (*r* = −0.15, *p* = 0.44), or Tsukamoto scores (*r* = 0.06, *p* = 0.75). Adjudicated performance scores were not significantly correlated with any subjective anxiety measures (*r* ≤ 0.28, *p* > 0.05). However, performance scores showed a significant positive correlation with cultural background (*r* = 0.55, *p* = 0.002), indicating systematic differences in adjudicated scores across cultural samples. Correlation coefficients for all variables are presented in Table 3.

**Table 3:** Pearson correlations among anxiety measures, physiological responses, performance scores, training condition, and cultural background.

Variable	1	2	3	4	5	6	7
1. STAI-Y1 (State anxiety)	1.00	0.58^***^	−0.02	0.82^***^	−0.07	−0.35	−0.26
2. SUDS	0.58^***^	1.00	−0.15	0.48^**^	−0.05	−0.18	0.04
3. HR change (ΔHR)	−0.02	−0.15	1.00	0.06	−0.22	−0.02	−0.31
4. Tsukamoto performance anxiety	0.82^***^	0.48^**^	0.06	1.00	−0.28	−0.31	−0.31
5. Performance score	−0.07	−0.05	−0.22	−0.28	1.00	0.09	0.55^**^
6. Training condition (VR = 1)	−0.35	−0.18	−0.02	−0.31	0.09	1.00	−0.00
7. Cultural background (Qiqihar = 1)	−0.26	0.04	−0.31	−0.31	0.55^**^	−0.00	1.00

STAI-Y1, State-Trait Anxiety Inventory, Form Y-1; SUDS, Subjective Units of Distress Scale; ΔHR, post-performance minus pre-performance heart rate; VR, virtual reality training condition. Cultural background was coded as Qiqihar University = 1, Tokyo University of the Arts = 0. *p < 0.05, **p < 0.01, ***p < 0.001.

### Regression analysis

To further examine the relationships among virtual reality training, cultural background, and anxiety-related measures, a series of multiple linear regression analyses were conducted. First, pre-performance state anxiety (STAI-Y1) was entered as the dependent variable, with training condition (VR vs. NVR), cultural background (Qiqihar University vs. Tokyo University of the Arts), and their interaction term included as predictors. The results indicated that the interaction between training condition and cultural background was significant (*B* = −20.18, *p* = 0.016), whereas the main effects of training condition (*p* > 0.05) and cultural background (*p* > 0.05) were not statistically significant. The overall model explained 36% of the variance (*R^2^* = 0.36).

Similarly, when post-performance subjective anxiety (Tsukamoto Performance Questionnaire score) was used as the dependent variable, the interaction between training condition and cultural background was also significant (*B* = −20.34, *p* = 0.005), while the main effects of training condition (*p* > 0.05) and cultural background (*p* > 0.05) did not reach significance. This model accounted for 41% of the variance (*R^2^* = 0.41).

A subsequent regression analysis was conducted with post-performance subjective anxiety (Tsukamoto score) as the outcome variable, including pre-performance state anxiety (STAI-Y1), subjective distress (SUDS), and heart rate change (ΔHR) as predictors, while controlling for training condition and cultural background. The results showed that STAI-Y1 was the only significant predictor (*B* = 0.66, *p* < 0.001). SUDS (*p* > 0.05), heart rate change (*p* > 0.05), training condition (*p* > 0.05), and cultural background (*p* > 0.05) were not significant predictors. The model explained 68% of the variance (*R^2^* = 0.68).

Finally, a regression analysis was performed with adjudicated performance scores as the dependent variable. The results indicated that cultural background was the only significant predictor (*B* = 4.84, *p* = 0.004), whereas pre-performance state anxiety (*p* > 0.05), subjective distress (*p* > 0.05), heart rate change (*p* > 0.05), and training condition (*p* > 0.05) did not reach statistical significance. This model accounted for 34% of the variance (*R^2^* = 0.34) (see Tables 4, 5).

**Table 4:** Multiple regression analyses predicting pre-performance state anxiety (STAI-Y1) and post-performance subjective anxiety (Tsukamoto Score).

Dependent variable	Predictor	B	SE	*p*
STAI-Y1 (State anxiety)	Intercept	49.00	3.76	< 0.001
	VR (0 = NVR, 1 = VR)	0.75	5.32	0.889
	Culture (0 = Tokyo, 1 = Qiqihar)	3.71	5.51	0.506
	VR × Culture	−20.18	7.79	0.016^*^
Tsukamoto anxiety (Post-performance self-evaluation)	Intercept	49.25	3.21	< 0.001
	VR (0 = NVR, 1 = VR)	2.63	4.55	0.569
	Culture (0 = Tokyo, 1 = Qiqihar)	3.32	4.71	0.487
	VR × Culture	−20.34	6.65	0.005^*^

VR, virtual reality training; NVR, Non-virtual reality condition. *p < 0.05.

**Table 5:** Multiple regression analyses predicting post-performance subjective anxiety (Tsukamoto Score) and Jury performance scores.

Dependent variable	Predictor	B	SE	*p*
Tsukamoto Anxiety (Post-performance self-evaluation)	Intercept	16.15	6.90	0.028
	STAI-Y1 (State Anxiety)	0.66	0.14	< 0.001^***^
	SUDS	0.32	0.79	0.688
	PR delta (Heart rate change)	0.03	0.09	0.715
	VR (0 = NVR, 1 = VR)	−0.91	2.73	0.743
	Culture (0 = Tokyo, 1 = Qiqihar)	−2.40	2.86	0.410
Jury Performance Score	Intercept	76.98	3.63	< 0.001^***^
	STAI-Y1 (State Anxiety)	0.08	0.08	0.290
	SUDS	−0.39	0.42	0.359
	PR delta (Heart rate change)	−0.02	0.05	0.744
	VR (0 = NVR, 1 = VR)	1.09	1.44	0.455
	Culture (0 = Tokyo, 1 = Qiqihar)	4.84	1.51	0.004^**^

STAI-Y1, State–Trait Anxiety Inventory (state form); SUDS, Subjective Units of Distress Scale; PR delta, post-performance minus pre-performance heart rate; VR, Virtual Reality training; NVR, Non-Virtual Reality condition. **p < 0.001, *p < 0.01.

### Summary of the results

To facilitate an overall view of the findings across outcome measures, Table 6 summarizes the statistical results for each dependent variable across the main analyses, including the effects of training condition, cultural background, and their interaction.

**Table 6:** Summary of main statistical findings across outcome measures.

Dependent variable/indicator	Primary analytical approach	Main effect of VR	Main effect of culture	VR × Culture interaction	Direction of findings
STAI-Y1 (Pre-performance state anxiety)	Two-way ANOVA; regression models	n.s.	n.s.	✓	Lower anxiety in the VR group within the Qiqihar sample
SUDS (Pre-performance subjective distress)	Two-way ANOVA; correlation analysis	n.s.	n.s.	n.s.	No clear or consistent differences observed
Tsukamoto performance anxiety (Post-performance self-evaluation)	Two-way ANOVA; regression models	n.s.	n.s.	✓	Lower anxiety in the VR group within the Qiqihar sample
Heart rate change (PR delta)	Two-way ANOVA; correlation analysis; regression models	n.s.	n.s.	n.s.	No stable or systematic differences observed
Adjudicated performance score (Objective performance)	Two-way ANOVA; regression models	n.s.	✓	n.s.	Higher scores observed in the Qiqihar sample

n.s., not significant.

## Discussion

The present findings indicate that virtual stage training was associated with significant reductions in pre-performance state anxiety (STAI-Y1) and post-performance subjective anxiety (Tsukamoto Performance Questionnaire) among Chinese students, whereas no comparable effects were observed among Japanese students. This interaction pattern is consistent with the argument advanced in the Introduction that music performance anxiety is not solely a physiological or psychological reaction, but an experience that may be shaped by broader sociocultural conditions. These findings should not be taken as definitive evidence of cultural moderation, but rather as preliminary indications that warrant further investigation.

The observed improvement among Chinese students may tentatively relate to differences in training context, particularly in how evaluative performance situations are experienced. However, these interpretations remain speculative, as the present study did not directly assess educational structures or institutional practices. Prior research has shown that Chinese music training is closely embedded in evaluation-oriented systems, including graded examinations, competitive ranking, and frequent performance-based assessment (Bao and Yu, 2025). Within such environments, anxiety may be closely tied to the experience of being observed and judged. The VR environment used in the present study, which incorporated realistic visual representations of adjudicators and audiences, closely approximated these external evaluative stressors. From this perspective, the VR intervention may be interpreted as a form of controlled exposure that allowed participants to rehearse under evaluative conditions within a psychologically manageable context (Bissonnette et al., 2016). When the primary source of anxiety appears to align with the form of intervention, opportunities for psychological adaptation may be enhanced.

By contrast, the pattern observed among Japanese students may points to a different pathway underlying performance anxiety. Despite exposure to the same immersive VR environment, Japanese participants did not show significant anxiety reduction. This finding is consistent with prior work emphasizing the role of shame, internalized responsibility, and relational norms in Japanese performance contexts (Hebert, 2012; Irie et al., 2023). For students studying in highly selective institutions such as Tokyo University of the Arts, anxiety may arise less from audience presence itself and more from concerns about failing to meet internal standards or violating perceived social expectations. Such forms of anxiety may be embedded in moral and relational frameworks and may not be readily modified through visual exposure alone. In this sense, while VR can reproduce the physical characteristics of a performance setting, it may not fully capture the psychological conditions required to engage culturally specific sources of anxiety in the Japanese context.

These findings do not suggest that VR-based interventions lack value for Japanese students. Rather, they suggest that cultural background may function as a moderating factor that shapes how individuals respond to intervention strategies. For performers whose anxiety appears to be primarily driven by external evaluation and competition, visual exposure may represent an effective approach. When anxiety appears to be influenced by internalized social norms or perfectionistic self-demands, VR-based interventions may require integration with reflective or cognitive components to more effectively address the underlying psychological mechanisms. Such differences may reflect not only cultural factors, but also variations in training background and practice intensity.

Although the present findings indicate that VR-based training was associated with reduced subjective anxiety in a subset of participants—most notably among Chinese students—this effect was not mirrored in the physiological data. Heart rate indices remained stable across training conditions and did not differ significantly as a function of cultural background. Notably, the pattern observed in the present data suggests that changes in perceived anxiety and physiological responses may follow different trajectories, rather than unfolding in a tightly coupled manner. This interpretation is consistent with earlier accounts in the anxiety literature, which have emphasized that experiential and physiological responses do not necessarily evolve in parallel (Rachman and Hodgson, 1974).

From this perspective, music performance anxiety may be better understood as a multi-layered state in which cognitive–emotional processes and physiological arousal are regulated through partially independent mechanisms. In the present study, VR training relied primarily on repeated visual exposure to performance-related contexts, allowing participants to become familiar with the stage environment and to cognitively reappraise its perceived threat. Such processes are likely to manifest first at the psychological level. Physiological arousal governed by the autonomic nervous system, by contrast, may be less responsive to short-term cognitive change or may remain elevated due to the sustained attentional and motor demands of musical performance. As a result, reductions in perceived anxiety do not necessarily entail corresponding changes in heart rate.

Instrument-specific demands may further contribute to this dissociation. Clarinet performance requires substantial breath control and regulation of thoracic pressure, both of which directly influence cardiovascular and respiratory activity. Relative to performance contexts involving keyboard instruments (e.g., Thompson et al., 2025), wind instrument performance imposes greater metabolic and respiratory load, potentially obscuring anxiety-related fluctuations in heart rate. Similar patterns have been reported in prior studies, which found that although VR-based interventions can improve subjective performance experience, corresponding physiological changes are often limited or inconsistent (Bissonnette et al., 2016).

Taken together, these findings suggest that, within the present experimental context, VR-based stage training predominantly influences the cognitive and experiential dimensions of music performance anxiety rather than physiological arousal per se. This underscores the importance of evaluating intervention outcomes using multiple indicators. The present findings also raise the question of how cognitive relief and physiological regulation might be more effectively aligned in music performance contexts. One possible direction may involve examining the role of physiological feedback or breathing-related regulation processes alongside immersive performance training.

The present study further showed that, although VR-based training was associated with reduced subjective anxiety in some participants, these changes were not accompanied by corresponding improvements in performance scores. Statistical analyses indicated that performance quality was more strongly related to the school factor than to training condition or measurement time. From this perspective, the association between anxiety and performance does not appear to follow a simple or proportional pattern. The observed dissociation indicates that performance execution is shaped by factors that are not readily modified by short-term changes in psychological state.

The findings of present study indicate that reductions in pre-performance anxiety do not necessarily translate into observable changes in performance execution. Although VR-based training was associated with lower levels of state anxiety among Chinese students, this effect appears to have been confined primarily to subjective experience and may not have reached a magnitude sufficient to influence motor execution or musical expressivity. One contributing factor may be the relative stability of performance execution shaped by long-term professional training. Under such conditions, highly practiced skills tend to operate with consistency and are less responsive to short-term fluctuations in psychological state.

With respect to performance scores, differences observed across schools should be considered in relation to broader educational contexts. Performance outcomes are shaped not only by individual proficiency, but also by long-standing features of training environments, including pedagogical approaches and assessment practices. Such factors tend to operate over extended periods of time and may therefore contribute to relatively stable performance patterns that are less responsive to short-term psychological interventions. Within this context, the present VR-based training was not intended to alter established technical habits or overall performance profiles. Rather, its effects appear to be more closely related to performers’ subjective experience during stage exposure, a tendency that has been noted in prior work examining immersive interventions in performance settings (Bissonnette et al., 2016).

Thus, these findings caution against viewing VR as a tool for rapid performance enhancement. The absence of significant effects on physiological responses and performance outcomes further suggests that the impact of the intervention may be limited or domain-specific, particularly within a short training period. Instead, its educational value may lie in supporting performers’ capacity to engage with high-pressure tasks under conditions of reduced psychological burden and greater subjective stability. For students experiencing persistent music performance anxiety, the ability to perform in a more regulated psychological state represents a meaningful outcome in its own right, even when short-term improvements are not directly reflected in adjudicated scores. Within professional music education, psychological adaptation and technical performance appear to develop along related but distinct trajectories.

### Limitations

Three limitations should be noted. First, the sample size was relatively small (*N* = 30), and only clarinet players were included. This was partly due to the specific recruitment criteria, as participants needed to be music majors preparing for formal performance examinations, and further constrained by the cross-cultural design involving two institutions. As a result, the pool of eligible participants was limited. While such sample sizes are not uncommon in studies with trained performers, the generalizability of the findings remains restricted. In addition, differences between instruments in physiological and motor demands mean that the present findings may not readily extend to other instrumental groups. Most participants were undergraduate students aged 18 to 24, reflecting an early stage of professional development. There were also differences between the two groups in years of training and practice intensity, which may have influenced anxiety responses and performance outcomes. These factors should be considered when interpreting the cross-cultural findings. Finally, the relatively short intervention period (5 days, with brief daily sessions) may have limited the extent of observable change, particularly for physiological responses and performance outcomes. Given the number of statistical tests conducted, the potential for inflated Type I error cannot be entirely ruled out, and the findings should be interpreted with appropriate caution. In addition, the regression models included interaction terms despite the relatively small sample size, which may have reduced model stability and increased the risk of overfitting. Therefore, the regression results should be interpreted with caution.

With regard to the technological implementation of virtual reality, this study focuses on visual realism, while simulation techniques for auditory feedback still require further development. For professional musicians, performance involves continuous interaction with auditory feedback, through which tone quality and dynamics are adjusted in response to the acoustic properties of the performance space. Discrepancies between virtual and real auditory feedback may thus influence fine-grained performance regulation, potentially limiting the extent to which VR-based training translates into immediate performance gains.

Finally, although culturally differentiated responses to VR training were observed between Chinese and Japanese students, both groups belong to the broader East Asian cultural context and share certain historical and cultural commonalities. This design allowed for a focused comparison of variations within collectivistic cultures—such as differences between externally evaluation-oriented pressure and more internalized, shame-related forms of anxiety—but it also constrains the generalizability of the findings to other cultural settings. Future studies incorporating participants from Western or other cultural backgrounds would help clarify how cultural factors moderate the effectiveness of technological interventions for music performance anxiety, thereby strengthening the external validity of this line of research.

## Conclusion

From a cross-cultural empirical standpoint, this study examined the role of virtual reality (VR) exposure training in the regulation of music performance anxiety. The present findings suggest that the effects of VR-based exposure training may not be uniform across participants and may vary depending on cultural and educational context. Within the present sample, participants from the Chinese group who received VR training reported lower levels of pre-performance state anxiety and post-performance uneasiness relative to the non-VR condition. By contrast, comparable anxiety-related differences were not clearly observed within the Japanese sample, although the underlying reasons for this pattern remain unclear. These results emphasize the importance of modulating MPA within specific cultural and educational contexts.

The present study also showed that reductions in subjective anxiety were not necessarily accompanied by corresponding changes in physiological responses or performance outcomes. Although VR training was associated with more favorable subjective evaluations of the performance situation in some participants, these patterns were not accompanied by clear differences in heart rate or adjudicated performance scores. This dissociation likely reflects the high level of technical stability characteristic of professionally trained music students, whose performance skills tend to remain robust despite short-term fluctuations in psychological state. Within the scope of the present study, VR-based training appeared to function more as a tool for supporting subjective adaptation to high-pressure performance situations than as a direct means of enhancing technical performance.

On this basis, the present study underscores the importance of cultural specificity in the application of technological interventions. When integrating VR-based training into music education, attention should be given to students’ cultural backgrounds and dominant sources of anxiety. In some contexts, VR exposure may need to be combined with approaches such as cognitive reappraisal or guided reflection, rather than relying on situational simulation alone. Moreover, the evaluation of intervention outcomes should distinguish between psychological adaptation and technical performance, which represent related but non-identical dimensions of development and should not be judged solely on short-term performance scores.

Future research may further examine integrative VR-based intervention models that incorporate physiological feedback, auditory feedback, or breathing regulation strategies, with the aim of exploring whether such approaches promote greater alignment between psychological and physiological responses. Expanding samples to include a wider range of cultural contexts and instrumental groups would also help to develop more context-sensitive approaches to addressing music performance anxiety across diverse educational settings.

## Data Availability

The raw data supporting the conclusions of this article will be made available by the authors, without undue reservation.
